# Editorial: Treatment resistant depression: epidemiology, clinic, burden and treatment, volume II

**DOI:** 10.3389/fpsyt.2026.1935707

**Published:** 2026-07-22

**Authors:** Vassilis Martiadis, Koen Demyttenaere, Giovanni Martinotti, Andrea Fiorillo

**Affiliations:** 1 Department of Mental Health, Asl Napoli 1 Centro, Naples, Italy; 2 Faculty of Medicine, University Psychiatric Center, KU Leuven, Leuven, Belgium; 3 Department of Neurosciences, Imaging and Clinical Sciences, Università degli Studi G. D’Annunzio Chieti-Pescara, Chieti, Italy; 4 Department of Psychiatry, University of Campania “L. Vanvitelli”, Naples, Italy

**Keywords:** bipolar depression, difficult-to-treat depression (DTD), neuromodulation, precision psychiatry, rapid-acting antidepressants, staging, treatment-resistant depression (TRD)

Treatment-resistant depression (TRD), including both unipolar and bipolar depression, remains a major clinical and public health challenge. Beyond the well-known operational definition based on failure of two successive adequate antidepressant trials, TRD is increasingly conceptualized as a dynamic, episode-spanning phenotype, shaped by symptom heterogeneity, comorbidity, and treatment history, and best captured through longitudinal staging and measurement-based care, or as Difficult-to-Treat Depression (DTD), which is defined as depression that continues to cause significant burden despite normal treatment efforts (1, 2).

Building on the first Research Topic dedicated to TRD epidemiology, clinical features, burden, and treatment (3), this Volume II aimed to consolidate multidisciplinary contributions spanning population-level burden, clinical characterization, and emerging pharmacological and non-pharmacological interventions.

**Figure 1:**
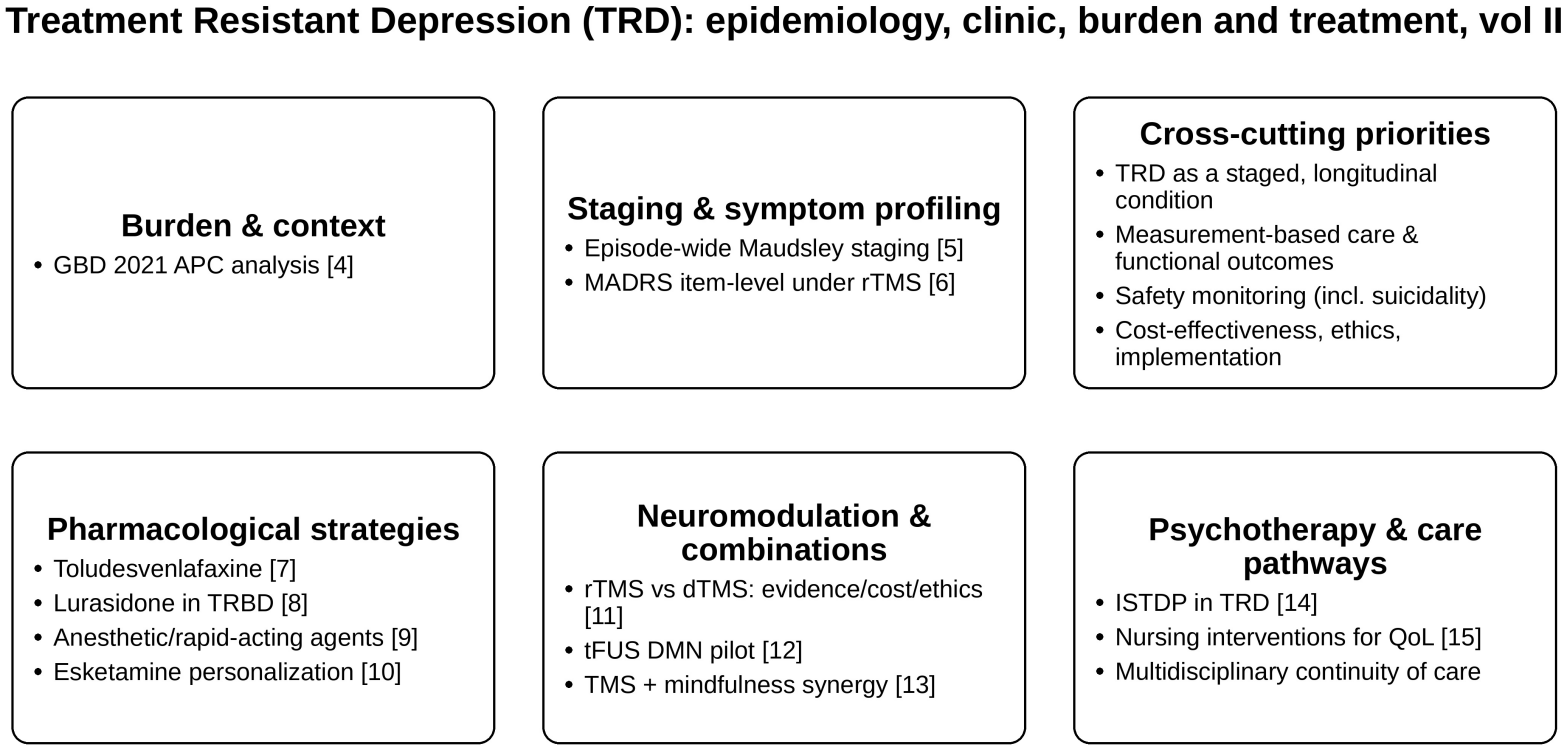
Overview of the main domains covered in this Research Topic, mapping the twelve contributions across: population-level burden and context (4); staging and symptom profiling (5, 6); pharmacological strategies (7–10); neuromodulation and combined approaches (11–13); and psychotherapy and care pathways (14, 15). Numbers in brackets correspond to the reference list.

The twelve articles collected in this Research Topic illustrate the need to approach TRD along a continuum: from upstream determinants and health-system constraints, to refined phenotyping and staging, to a broader therapeutic armamentarium that includes optimization of antidepressant strategies, rapid-acting agents, neuromodulation, and psychosocial care, while keeping functional outcomes and quality of life as central endpoints (**Figure 1**).

A population-level perspective is provided by Zhang et al., who used Global Burden of Disease 2021 data to compare major depressive disorder burden across China, India, and the United States through an age–period–cohort framework. Their analysis highlights how prevalence and years lived with disability are shaped by distinct demographic transitions and period effects, underscoring that the “burden context” in which TRD develops and is treated differs markedly across settings. These findings strengthen the rationale for aligning TRD research and service delivery with local epidemiology, resources, and policy priorities rather than assuming cross-national transferability.

At the individual clinical level, Antoniou et al. address a key methodological gap: how to quantify resistance meaningfully over time. By testing an episode-wide application of the Maudsley Staging Method in a longitudinal tertiary-care cohort, they compare dimensional and categorical outcomes and demonstrate that staging can capture clinically relevant gradients of resistance. Their findings support a shift from dichotomous definitions toward staging-informed stratification, which may be valuable for prognosis, trial enrichment, and next-step intervention selection in complex, multi-failure pathways.

A complementary contribution by Viegas et al. speaks directly to measurement-based care and symptom heterogeneity. In an exploratory study of rTMS response, the authors examine changes in both total and item-level Montgomery–Åsberg Depression Rating Scale (MADRS) scores in patients with melancholic versus “unspecified” depressive features. By suggesting differential trajectories across subtypes, and highlighting that specific symptom domains may improve unevenly, this work reinforces the clinical importance of moving beyond global severity scores to symptom-level monitoring, particularly when neuromodulatory interventions are considered.

Several contributions expand the pharmacological landscape across different points of the resistance continuum. Yang et al. report a multicenter, single-arm clinical study of toludesvenlafaxine sustained-release tablets in patients with depression who had an ineffective or only partially effective response to an initial antidepressant. In a real-world-relevant scenario of early non-response or partial response, they document symptomatic improvement alongside an acceptable safety profile, reinforcing the importance of timely treatment optimization before resistance becomes entrenched.

Porceddu et al. on treatment-resistant bipolar depression (TRBD), an area where evidence is often extrapolated from unipolar TRD despite meaningful pathophysiological and therapeutic differences. Their retrospective observational analysis provides pragmatic information on real-world outcomes and tolerability of lurasidone augmentation, supporting more data-driven decision-making when atypical antipsychotic strategies are considered in bipolar depression with inadequate response.

A broader mechanistic and translational view of rapid-acting approaches is offered by Li et al., who review novel and emerging anesthetic agents with antidepressant potential. By integrating evidence on ketamine and esketamine with data on other anesthetic candidates, including propofol and inhalational agents such as nitrous oxide, sevoflurane, and isoflurane, the authors outline shared and divergent molecular targets and discuss practical barriers to implementation (e.g., safety monitoring, durability of effects, and scalability). This synthesis is timely as the field seeks to translate rapid symptom relief into sustained recovery strategies.

Personalization of rapid-acting treatment is further advanced by Olivola et al., who examine psychological dimensions, such as mentalization, cognitive rigidity, psychache, and suicidality, in patients with TRD and TRBD receiving esketamine. Their findings highlight the value of integrating clinical phenomenology and patient-reported psychological constructs with treatment delivery, not only to understand heterogeneity of response but also to prioritize monitoring of suicide-related outcomes in real-world settings.

Neuromodulation is represented through both critical appraisal and innovation. Paganin addresses a translational paradox: the widespread clinical use of both repetitive transcranial magnetic stimulation (rTMS) and deep TMS (dTMS) in TRD despite a limited head-to-head comparative evidence base. By discussing regulatory pathways, the absence of robust cost-effectiveness data, and the ethical implications of decision-making under uncertainty, this contribution sets out research priorities that include comparative effectiveness trials, connectivity-informed targets, and predictive models that integrate multimodal clinical and neurobiological data.

Complementing this perspective, Schachtner et al. report an open-label pilot trial of non-invasive transcranial focused ultrasound (tFUS) for depression, targeting default mode network (DMN) hyperconnectivity—a network-level mechanism linked to repetitive negative thinking. Their work provides early evidence that tFUS can engage DMN-related connectivity metrics alongside clinical and cognitive symptom measures, illustrating a mechanism-driven direction for precision neuromodulation.

Moving from device selection to multimodal optimization, Yun and Figee propose a neurobiological rationale for combining TMS with brief, adjunctive mindfulness-based interventions. Grounded in the triple-network model, their mini-review frames mindfulness as a potential “behavioral primer” that may shift large-scale network states (DMN, salience, and central executive networks) to enhance state-dependent plasticity and possibly reduce variability in rTMS response. This contribution aligns with a broader shift toward interventional strategies that integrate exogenous neuromodulation with endogenous self-regulation skills, and it highlights the need for trials that test dose, timing, and mechanistic biomarkers.

Psychological and multidisciplinary care are also central to durable recovery in TRD. Johansson and Lilliengren reanalyze data from a randomized controlled trial of Intensive Short-Term Dynamic Psychotherapy (ISTDP) for TRD, confirming large and durable symptom improvements. Importantly, their mediation and cross-lagged analyses do not support the theorized sequential mechanisms of change; instead, the intervention appears to produce broad, concurrent improvements across multiple psychological domains. These findings encourage more fine-grained, temporally sensitive measurement of in-session processes to better understand how psychotherapy exerts its effects in treatment-resistant populations.

Finally, Liao et al. broaden the scope beyond symptom reduction by reviewing nursing interventions aimed at improving quality of life in major depressive disorder. By mapping interventions such as psychoeducation, case management, cognitive-behavioral and mind–body techniques, and social reintegration support, the review emphasizes continuity of care as a therapeutic ingredient, particularly relevant for chronic, relapsing courses where functional recovery depends on sustained and multidisciplinary engagement.

Taken together, the contributions in this Research Topic converge on several priorities for the next phase of TRD research and care. First, TRD should be operationalized as a staged, longitudinal condition, supported by systematic assessment of symptom domains, functioning, and suicide-related risk. Second, expanding the therapeutic toolbox—spanning optimized antidepressant strategies, augmentation in bipolar depression, anesthetic-derived rapid-acting agents, and emerging neuromodulation—must be matched by rigorous comparative effectiveness research, including health-economic evaluation and equity-informed implementation. Third, personalization will likely require integrating clinical staging, psychological constructs, and neurobiological markers to better match patients to interventions and to design sustainable maintenance pathways.

We hope that this Volume II Research Topic will stimulate further cross-disciplinary work linking epidemiology, mechanistic neuroscience, psychotherapy research, and pragmatic clinical studies, ultimately improving outcomes for individuals living with TRD and TRBD.
